# Metastatic adenocarcinomas of left colon and rectum: differences in clinical outcome and gene expression

**DOI:** 10.3389/fonc.2026.1797579

**Published:** 2026-04-15

**Authors:** N. N. Semenov, L. G. Zhukova, V. A. Yakovlev

**Affiliations:** 1 State Budgetary Institution of Healthcare (SBIH) A.S. Loginov Moscow Clinical Scientific and Practical Center of the Department of Healthcare of the City of Moscow, Moscow, Russia; 2 Massey Comprehensive Cancer Center, Virginia Commonwealth University (VCU) School of Medicine, Richmond, VA, United States

**Keywords:** anti-VEGF therapy, chemotherapy, gene expression, left-sided colon cancer (LSCC), metastatic colorectal cancer (mCRC), rectal cancer (RC), TCGA database

## Abstract

**Background:**

There are relatively well-substantiated concepts regarding the differences in the clinical course of metastatic colorectal cancer depending on the primary tumor localization: right- and left-sided (including rectal). However, many clinical trials demonstrate clear differences in the selected efficacy of adjuvant chemotherapy in the treatment of rectal versus colon cancer. We hypothesize that there are significant differences in long-term outcomes between patients with metastatic rectal cancer (RC) and those with left-sided colon cancer (LSCC) that may depend on the genetic profile of the primary tumor.

**Methods:**

155 patients with synchronous metastases of LSCC and 96 patients with RC were selected for clinical analysis. The TCGA database was used to obtain information on mRNA expression from the “Comprehensive molecular characterization of human colon and rectal cancer” study. 153 patients with LSCC and 41 patients with RC were included in this analysis.

**Results:**

Clinical analysis characterized by a significantly more favorable course of the disease after I and I+II lines of chemotherapy for RC group, which may be associated with a higher expression of EBI3, IL-17A, and SOCS1 in the primary tumor. Also, the RC group is characterized by a higher sensitivity to anti-VEGF therapy, which can be explained by a higher expression of VEGF-A, VEGF-D, and KDR-VEGFR2 compared to the LSCC group.

**Conclusion:**

Our study shows that when analyzing the effectiveness of antitumor therapy in patients with colorectal cancer, it is necessary to distinguish not only between groups with cancer of the right and left colon, but also to consider rectal cancer as a third independent group.

## Introduction

Currently, there are relatively well-substantiated concepts regarding the differences in the clinical course of metastatic colorectal cancer that depend on primary-tumor localization – right-sided (cecum, ascending colon, transverse colon) and left-sided (descending colon, sigmoid colon, rectosigmoid junction, and rectum) (1–3). This distinction is rooted in different ontogenesis (in embryogenesis, the proximal colon originates from the midgut, whereas the distal colon derives the hindgut), different pathways of carcinogenesis, and consequently, different mutation rates of oncogenes. It has been shown that when metastases develop, the prognosis in right-sided localization is worse with both chemotherapy and chemotherapy + anti-VEGF drugs than in left-sided localization. Moreover, the use of anti-EGFR therapy for right-sided colon cancer has no advantage over chemotherapy alone, and therefore it is not recommended in the first line of treatment (4). Because rectal cancer is located in the left colon, treatment recommendations for patients with metastatic rectal and left colon cancer do not differ. Hence, adjuvant chemotherapy for stage II-III rectal cancer is recommended, mainly by extrapolating data from colon-cancer trials. However, numerous clinical trials demonstrate clear differences in adjuvant chemotherapy efficacy in the treatment of rectal and colon cancer. For stage II-III rectal cancer in has been shown that the use of adjuvant chemotherapy in addition to neoadjuvant radiation therapy with concurrent use of 5-FU/Capecitabine, does not improve overall survival (PROCTOR-SCRIPT (5), EORTC 22921 (6), I-CNR-RT (7), CHRONICLE (8)); this contrasts with the survival benefit seen with Oxaliplatin plus fluoropyrimidines in stage III colon cancer (MOSAIC (9), NSABP C-07 (10), XELOXA (11)).

It is well known that the lower sections of the rectum have a unique venous outflow, which largely accounts for a different metastatic pattern compared to colon cancer (rectal cancer more frequently metastasizes to the lungs and less often to the peritoneum).

There are few studies that compare mutation frequencies and genetic profiles between left-sided colon cancer (LSCC, tumor localization from the splenic flexure to the rectosigmoid junction) and rectal cancer (RC). For example, J. Marshall et al. (2017) (12) reported that, compared with tumors of the splenic flexure and descending colon, rectal tumors have more frequent TP53 and APC mutations and less frequent PIK3CA, BRAF, GNAS, and HNF1A mutations. RC is characterized by higher expression of TOPO1, ERCC1, and MGMT than LSCC, and higher expression of TLE3, TOPO1, TUBB3, and MGMT comparing with sigmoid tumors (12). Microsatellite instability (MSI) was noted in 7% of tumors of the left flexure and descending colon, 4% of the sigmoid colon, and 0.7% of the RC (12).

Other studies indicated that, the frequency of MSI is 9-13% in LSCC and 4-5% in RC (13, 14). Interestingly, in LSCC patients, the presence of MSI conferred better recurrence-free and overall survival, whereas in RC patients it did not (14).

Thus, we hypothesize that long-term outcomes differ significantly between RC and LSCC overall and according to treatment received. We further hypothesize that the difference in clinical outcomes between RC and LSCC are driven by distinct genetic profiles.

### Objectives

The present study evaluates progression-free survival in I and I+II treatment lines and overall survival in patients with metastatic LSCC and RC groups of patients. We also compared genetic profiles of primary left-colon and rectal tumors using data from The Cancer Genome Atlas (TCGA).

## Materials and methods

### Patients

For the retrospective study, a total of 622 patients with metastatic cancer of the LSCC and 354 patients with RC were extracted from the unified medical database of the A.S. Loginov Moscow Clinical Scientific and Practical Center. Patients with palliative removal of the primary tumor, surgical treatment of metastases, metachronous metastases, and those receiving immune checkpoint inhibitors were excluded. As a result, 155 patients with synchronous metastases of LSCC and 96 patients with RC were selected for analysis. All patients received conventional treatment (Oxa and/or Iri + fluoropirimidines ± anti-EGFR or anti-VEGF). The treatment was carried out in 2015-2023. The median follow-up was 27.8 months (13.4–116 months). The data cut-off was in February 2025. The characteristics of the patients are presented in **Supplementary Table 1**.

### Statistical analysis of clinical data

The SPSS Statistics software version 26 was used for statistical analysis of clinical data. The average values (age, CEA level, and CA 19-9) were compared by the t-criterion for independent variables using the Levigne criterion. Nonparametric data were analyzed using the χ2 test or the Fisher criterion, depending on the number of observations. Survival was calculated by the Kaplan–Meier method, differences were assessed by a log-rank test; Hazard ratio (HR) and 95% confidence interval (CI) were indicated for the median survival. Differences were considered statistically significant at p-value <0.05. Progression–free survival (PFS) was calculated as the time from the start of antitumor treatment to disease progression, death, or last follow-up, and overall survival (OS) – was calculated to the date of death or last follow-up. Univariate and multivariate analyses of overall survival were conducted using Cox regression (Proportional Hazards Model) to assess the independent prognostic significance of clinical and molecular variables.

### The cancer genome atlas database analysis

The Cancer Genome Atlas (TCGA) database was used to obtain information regarding mRNA expression from the “Comprehensive molecular characterization of human colon and rectal cancer” study (15). The “rectum” group and the “left-side colon” group which includes the following localizations ‘descending colon’, ‘sigmoid colon’, and ‘splenic flexure’ were stratified. The data included expression of >28,000 genes with STAR counts. The data underwent pre-processing including data wrangling, and data normalization. This step included variance stabilizing transformation (vst) and Deseq2 package normalization to ensure data comparability across samples. For normalized mRNA data, multiple testing correction was applied using Benjamini-Hochberg (BH) false discovery rate method with a significance threshold set at p < 0.05. Stage-stratified analyses were conducted as exploratory subgroup analyses to further evaluate potential differences in gene expression patterns and clinical outcomes across disease stages. These analyses were not pre-specified in the initial study design and should therefore be interpreted as hypothesis-generating. The mRNA expression data were extracted, cleaned, normalized, analyzed, and presented with an original R-script. The characteristics of the patients are presented in **Supplementary Table 2**.

## Results

In general, the groups of LSCC and RC patients were well balanced, except for indicators that reflect tumor biology: the RC group had more frequent lung metastases, whereas the LSCC group had more frequent peritoneal metastases and higher levels of CEA and CA 19-9. Interestingly, patients with lung-only metastases were equally common in both groups. The mutation rates of BRAF and MSI did not differ. The use of targeted therapy was also distributed evenly between the RC and LSCC patient groups. Anti–EGFR drugs (cetuximab and panitumumab) were used only in RASwt cases. Anti-BRAF therapy was used in the III and IV lines of treatment for only 2 patients with LSCC and the corresponding mutation.

A univariate analysis of factors potentially influencing overall survival (OS), including sex, age >65 years, presence of lung or peritoneal metastases, ECOG performance status, primary tumor location, number of metastatic organs, and serum levels of CEA and CA 19–9 was performed. The analysis demonstrated a significant impact of age >65 years, ECOG performance status, and primary tumor location on OS (**Supplementary Table 3**). In the subsequent multivariate analysis, all of these factors remained statistically significant (**Supplementary Table 4**). However, no significant differences between the study groups were observed with respect to age (>65 years: 55.5% vs. 45.8%, p = 0.15) or ECOG performance status (ECOG 2: 12.9% vs. 7.3%, p = 0.20). Therefore, the groups were well balanced with regard to these characteristics.

### Comparison of progression-free survival of I and I+II lines of therapy and overall survival for LSCC and RC groups of patients

**Figure 1:**
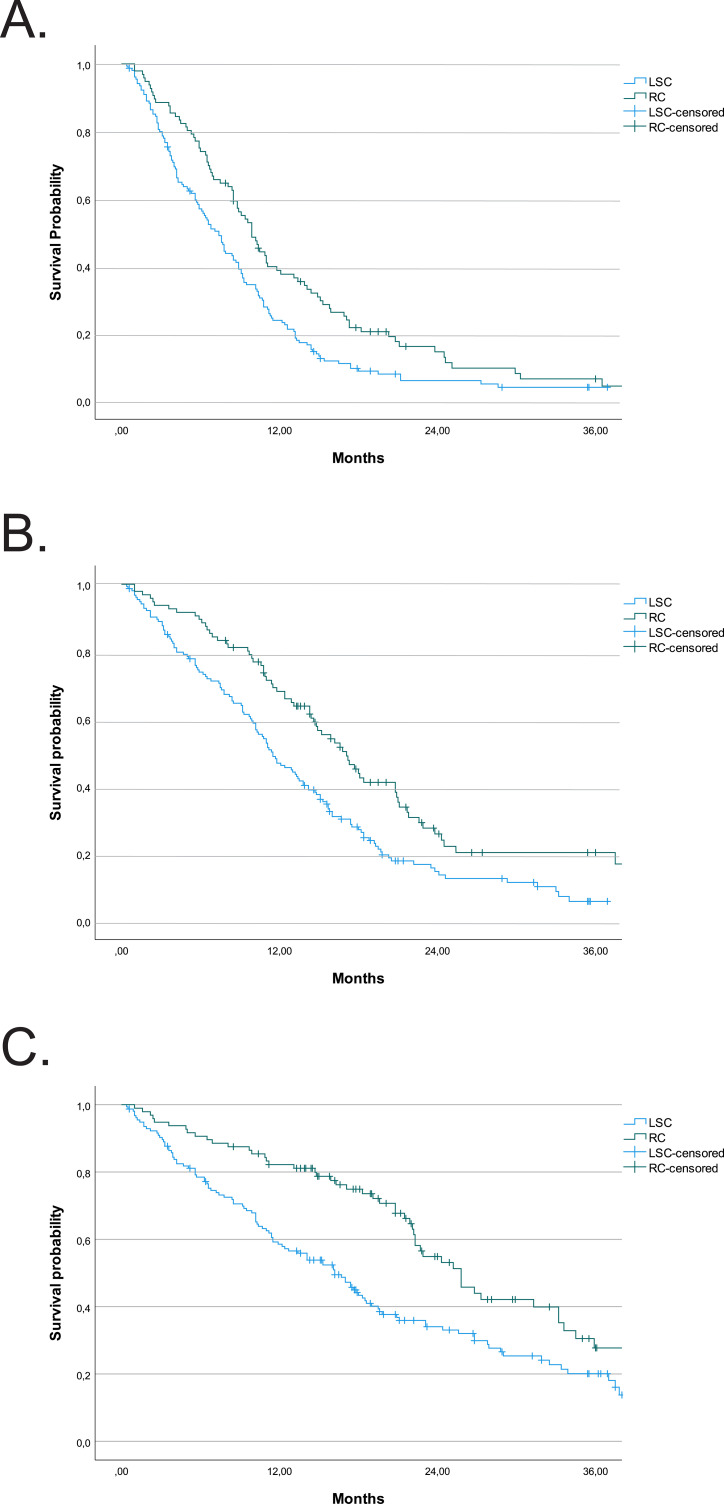
PFS and OS after I and I+II lines of therapy for LSCC and RC groups of patients. **(A)** PFS after I line of therapy. **(B)** PFS after I+II lines of therapy. **(C)** OS after I+II lines of therapy.

The availability of second-line treatment significantly increased PFS in both patient groups (**Supplementary Table 5**, **Figures 1A, B**). However, drug control of tumor growth was more effective in the RC patient group, which led to significantly longer PFS in both I line and I+II lines of treatment, as well as significantly longer OS for RC patients (**Supplementary Table 5**, **Figure 1C**).

### Impact of the “targeted” drugs on the PFS and OS after the I+II lines of chemotherapy

It has been shown that the addition of anti-VEGF (bevacizumab in I and II lines, aflibercept and ramucirumab in II lines) (16–22) and anti-EGFR (cetuximab and panitumumab in RASwt in I or II line of therapy) (23–25) to cytostatics can improve both PFS and OS. Since the use of targeted therapy is also recommended in the II line of anticancer therapy, we assessed the long-term results in accordance with their use in both lines of treatment. The analysis included a group that received anti-EGFR in the I line, then anti-VEGF in the II line (LSCC = 24 and RC = 19), or vice versa (LSCC = 9 and RC = 2) (**Table 1**).

**Table 1:** Progression-free survival and overall survival after the I+II lines of chemotherapy +/- targeted drugs.

Therapy	LSCC		RC		P-value
	n	PFS	n	PFS	
Chemo only	26	3.9 m.	11	6.5 m.	0.22 HR 1.63 95%CI 0.74-3.56
Chemo + anti-VEGF	64	11.5 m.	42	17.3 m.	0.09 HR 1.85 95%CI 1.16-2.94
Chemo + anti-EGFR	32	8.5 m.	22	11.0 m.	0.18 HR 0.64 95%CI 0.34-1.23
Chemo + anti-VEGF and anti-EGFR	33	8.9 m.	21	9.9 m.	0.32 HR 0.75 95%CI 0.43-1,32
Overall survival					
Chemo only	26	3.9 m.	11	6.5 m.	0.48 HR 1.31 95%CI 0.61-2.81
Chemo + anti-VEGF	64	17.4 m.	42	25.2 m.	0.018 HR 1.84 95%CI 1.11-3.06
Chemo + anti-EGFR	32	11.5 m.	22	27.3 m.	0.18 HR 0.61 95%CI 0.29-1.27
Chemo + anti-VEGF and anti-EGFR	33	19.6 m.	21	39.6 m.	0.004 HR 0.28 95%CI 0.12-0.66

When evaluating the results of I+II lines treatment, the best PFS results were observed in both groups of patients when chemotherapy was carried out in combination with anti-VEGF drugs (**Table 1**). At the same time, the PFS value in the RC patient group was considerably higher than in the LSCC group, although it did not reach a statistically significant difference (17.3m vs 11.5m, p-value=0.09). When evaluating overall survival, the combination of chemotherapy + Anti-VEGF also demonstrated significantly better efficacy for RC group of patients (25.2m vs 17.4m, p-value=0.018). However, if it was possible to consistently use both types of targeted therapy, then patients with RC demonstrated the longest OS. Interestingly, when using both targeted drugs in combination with chemotherapy, the difference in PFS between the LSCC and RC groups was practically absent (8.9m vs 9.9m, p-value=0.32), but the OS duration for the RS group was two-fold higher comparing to the LSCC group (39.6m vs 19.6m, p-value=0.004).

### Comparison of PFS and OS in patients with right-sided colon cancer, LSCC, and RC

**Figure 2:**
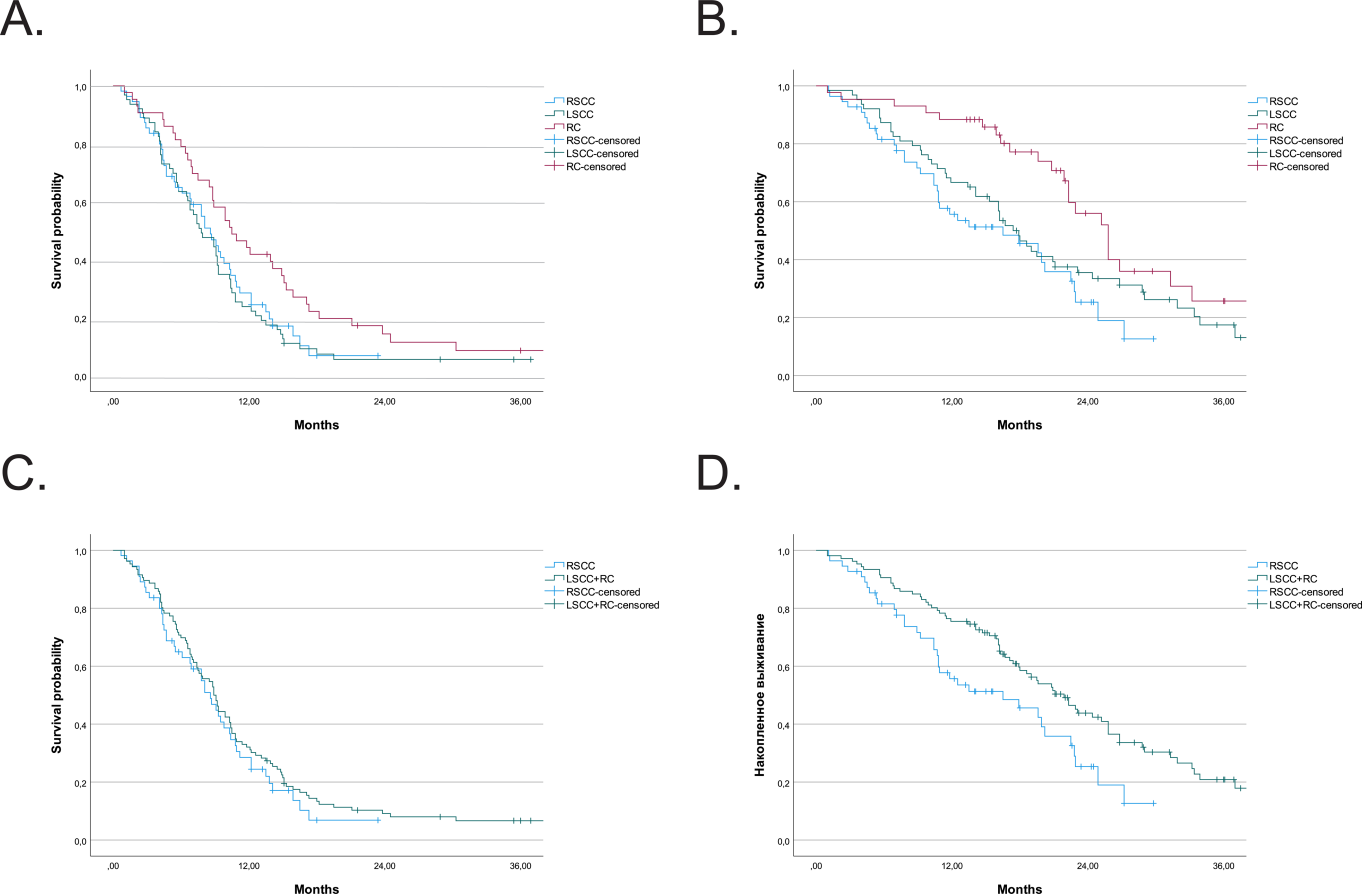
PFS and OS after I line chemotherapy for different tumor localization. **(A)** PFS for SCC, LSCC, and RC. **(B)** OS for RSCC, LSCC, and RC. **(C)** PFS for RSCC vs LSCC+RC groups. **(D)** OS for RSCC vs LSCC+RC groups.

Since the anti-EGFR therapy is not used for the RSCC patients, we compared the results of chemotherapy + anti-VEGF as the I line of the RSCC, LSCC, RC, and LSCC+RC patients (**Supplementary Table 6**, **Figure 2**). All patients had synchronous metastasis with no surgery to the primary tumor and metastasis. Anti-VEGF has previously been shown to be more effective in treatment of LSCC metastases than RSCC. However, there had been no independent analysis for RC group (26). In this regard, we found it important to compare the RC and LSCC groups with the RSCC group of patients who had similar characteristics. As **Supplementary Table 6**; **Figure 2** show, the better prognosis for left-sided colorectal cancer is provided by the RC patient group.

### TCGA database analyses for gene expression in the primary tumor of LSCC and RC groups

To analyze genetic differences between primary rectal and left colon tumors, we used mRNA expression data from the “Comprehensive molecular characterization of human colon and rectal cancer” study within the TCGA database (15). **Supplementary Table 2** demonstrates the characteristics of the patients used for the gene expression analysis.

**Figure 3:**
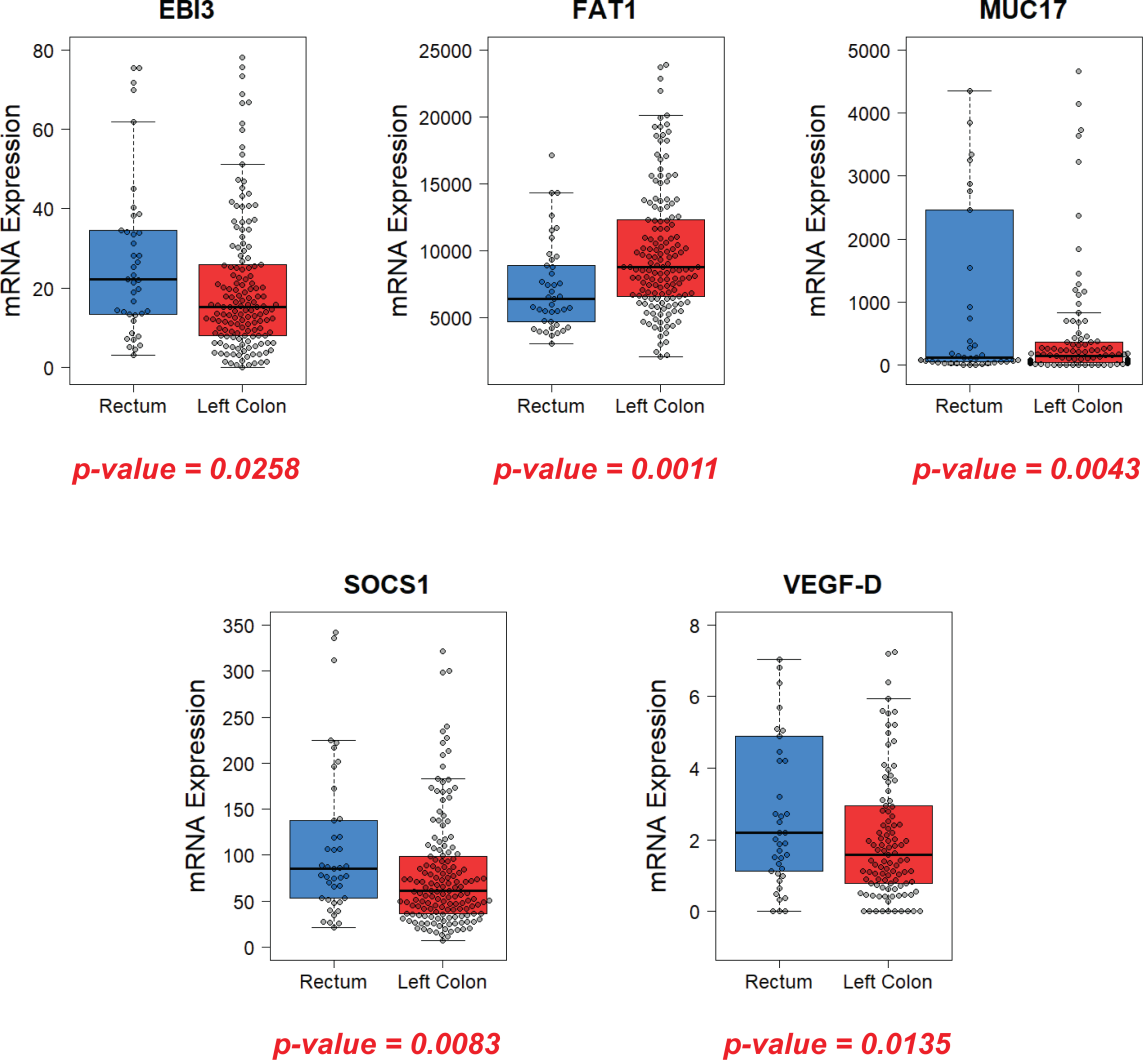
Comparison of gene expression in the primary tumor for the RC and LSCC groups of patients with all disease stages (I-IV). Genes with statistically significant differences (p-value < 0.05) are shown.

First, we compared LSCC and RC groups that included patients with all stages of the disease. Despite the fairly similar genetic expression pattern, a number of genes exhibited significant differences in mRNA expression (**Figure 3**).

The Epstein-Barr virus-induced gene 3 (EBI3) is a member of the interleukin-12 (IL-12) family structurally homologous to the IL-12p40 subunit and forms a heterodimer either with the IL-27p28 subunit to build IL-27 or with IL-12p35 subunit to form IL-35 (27). In an analysis of the TCGA database, EBI3 was found to exhibit significantly higher expression in the primary tumor of patients in the RC group compared with the LSCC group (median = 23.19±23.74 vs 15.18±19.86 respectively; p-value = 0.0258).

FAT1 is an atypical cadherin, a member of the FAT cadherin superfamily of membrane proteins containing cadherin repeat sequences (28). FAT1 plays a dual role in different cancers via the Hippo, Wnt, and MAPK/ERK signaling pathways (29–31). Analysis of TCGA database demonstrated that LSCC group demonstrated significantly higher expression of FAT1 compared with the RC patient group (median = 8761.39±5087.72 vs 6396.28±3338.19 respectively; p-value = 0.0011).

Mucin-17 (MUC17) is a transmembrane protein that is expressed in the normal colon and small intestine (32, 33). MUC17 is a member of a family of glycoproteins involved in cytoprotection, extravasation during metastases, maintenance of luminal structure, and signal transduction during colorectal cancer development (34). Analysis of the TCGA database demonstrated that hyperexpression of MUC17 is more common in the LSCC group compared with the RC group of patients (median = 145±1691.11 vs 120.74±5483.86 respectively; p=0.0043).

Suppressors of cytokine signaling (SOCS) proteins are modulators of cytokine and growth factor signaling whose aberrant regulation has been linked to regulation of growth, proliferation, and invasion of the different types of tumors (35–39). Among them, SOCS1 has been extensively investigated. Initially, it was recognized as a negative feedback regulator of cytokine signaling, particularly the Janus kinases–signal transducers and activators of transcription (JAK–STAT) signaling pathway, which is crucial for cellular activation, proliferation, and differentiation (40). SOCS1 Analysis of TCGA database demonstrated that RC patients demonstrate significantly higher expression of SOCS1 compared to the LSCC group (median = 85.18±83.64 vs 61.13±61.09 respectively; p=0.0083).

VEGF (Vascular Endothelial Growth Factor) is a family of proteins responsible for neoangiogenesis. The role of VEGF-A, which plays a key role in vascular neoplasia that promotes tumor growth and metastasis, is best understood. Agents that bind VEGF-A in the bloodstream (bevacizumab, aflibercept) and those that block receptors for VEGF (ramucirumab) have demonstrated efficacy in metastatic colorectal cancer (41–43). VEGF-D is less widely studied; it is known to be mainly responsible for a lymphangiogenesis. However, it was shown that when VEGF-A was suppressed (e.g., by anti-VEGF therapy), the synthesis of VEGF-D increased significantly and began to stimulate blood vessel growth more actively than VEGF-A (44). TCGA database analysis revealed that RC patients demonstrate significantly higher expression of VEGF-D in primary tumor compared to LSCC patients (median = 2.18±16.2 vs 1.57±4.24 respectively; p-value=0.0135).

Given that patients with advanced-stage colorectal cancer represent the primary population eligible for systemic chemotherapy and targeted therapies, we performed a stage-stratified gene expression analysis comparing RC and LSCC patients. Specifically, patients were separated into early-stage (I–II stages) and advanced-stage (III–IV stages) disease groups to evaluate both intergroup differences and stage-dependent transcriptional changes. Due to the limited number of RC cases available in the TCGA cohort, aggregation of stages into these broader categories was necessary to ensure sufficient statistical power.

**Figure 4:**
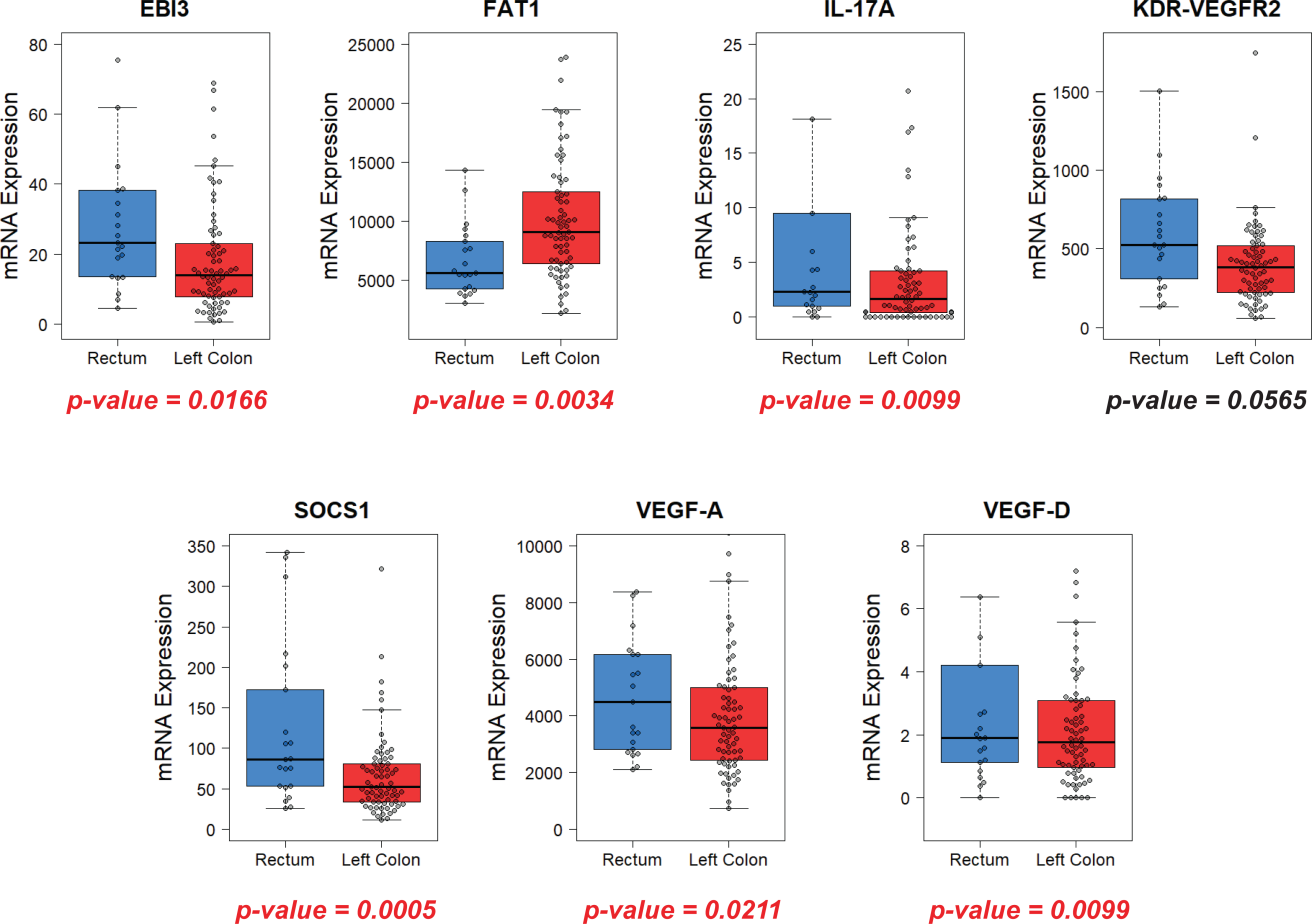
Comparison of gene expression in the primary tumor for the RC and LSCC groups of patients with the stages III-IV.

**Figure 5:**
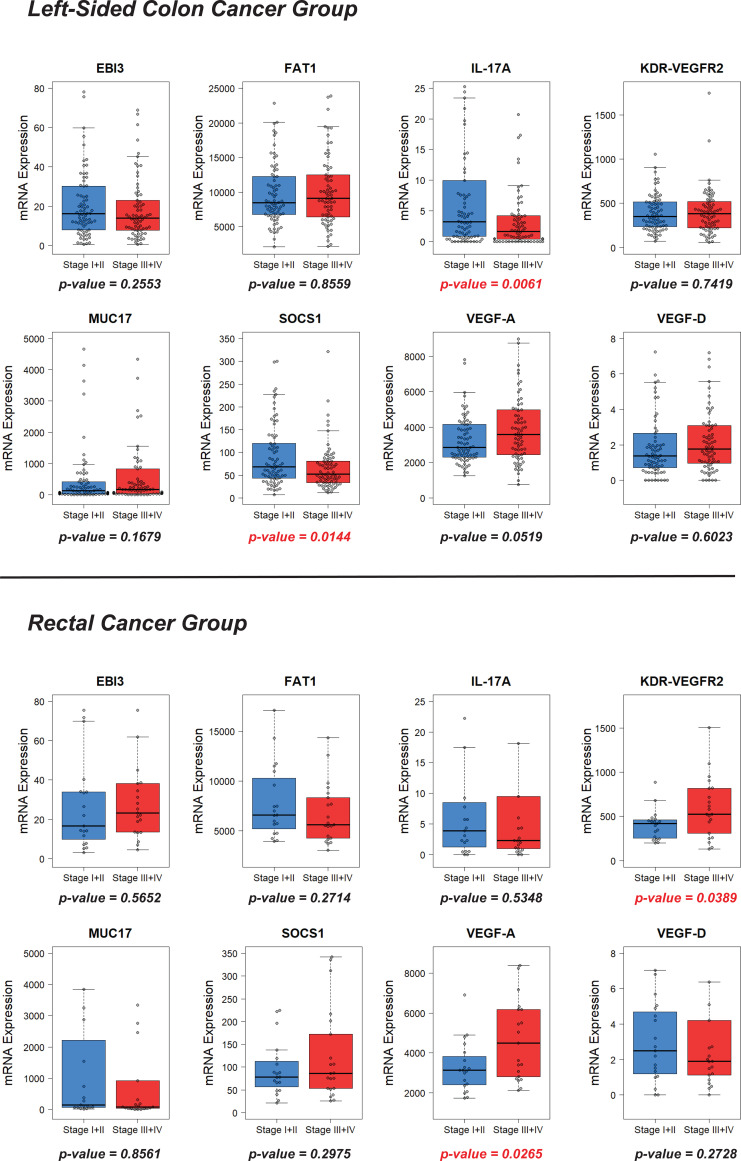
Changes in gene expression in the primary tumor depending on the stage of the disease (I-II vs III-IV) in the RC and LSCC groups.

Next, we analyzed gene expression for the LSCC and RC groups for the patients with stages III-IV of the disease, since these patients are the primary candidates for chemotherapy and targeted therapy (**Figure 4**). As expected, increasing disease stage leads to significant changes in the expression of certain genes in both groups of patients (**Figure 5**). Consequently, the results of gene expression analysis in the RC and LSCC groups for patients with stages III-IV are different from the analysis including patients across all stages. In the RC group, the expression of MUC17 decreases with advancing disease stage, while in the LSCC group it increases (**Figure 5**). Although these changes are not statistically significant, they eliminate the stage III–IV difference in MUC17 expression between the RC and LSCC groups. The expression of the genes EBI3, FAT1, and VEGF-D also demonstrates minor fluctuations in both groups depending on the stage of the disease (**Figure 5**). However, they all retain a statistical difference in expression between the RC and LSCC groups for patients with stages III-IV: for EBI3 – median expression 23.22±25.19 vs 13.94±15.77 respectively (p-value=0.0166); for FAT1 – median expression 5623.21±3004.37 vs 9090.29±5046.95 respectively (p-value=0.0034); for VEGF-D – median expression 1.9±22.32 vs 1.75±2.52 respectively (p-value=0.0099). SOCS1 showed a significant decrease in expression at higher disease stage in the LSCC group (p-value = 0.0144) (**Figure 5**). At the same time, in the RC group, with an increase in the stage of the disease, the expression of SOCS1 rises modestly (**Figure 5**). As a result, the difference in SOCS1 expression between the RC and LSCC groups widens further even more for patients with stages III-IV (median = 86.18±101.15 vs 52.13±51.02 respectively; p-value=0.0005). In the LSCC group, patients with more advanced disease stage demonstrated significantly reduced expression of interleukin 17A (IL-17A) in the primary tumor (p-value = 0.0061) (**Figure 5**). This decrease in expression leads to the fact that the difference in IL-17A expression between the RC and LSCC groups for patients with stages III-IV also becomes statistically significant median = 2.27±15.43 vs 1.63±6.62 respectively; p-value=0.0099) (**Figure 4**). Interestingly, the expression of VEGF-A and VEGF receptor KDR-VEGFR2 in the primary tumor was significantly increased in patients with more advanced stage of disease in the RC group (p-value = 0.0265 and 0.0389 respectfully) (**Figure 5**). The difference between the RC and LSCC groups for patients with stages III-IV becomes statistically significant for VEGF-A (median expression 4496.81±3318.8 vs 3581.47±2066.57 respectively; p-value=0.0211) and almost reach the level of statistical significance for KDR-VEGFR2 (median expression 525.76±343.22 vs 385.84±260.01 respectively; p-value=0.0565).

Thus, it was shown that the primary tumors of the LSCC and RC possess distinct baseline genetic profiles. The genetic profile of RC and LSCC groups may vary depending on the stage of the disease. Patients with the stage III-IV in the RC group exhibit significantly higher expression of the EBI3, IL-17A, SOCS1, and genes associated with tumor vascularization: VEGF-A and VEGF-D. Patients with the stage III-IV LSCC display significantly higher expression of the FAT1 gene.

## Discussion

Differences in long-term outcomes between right- and left-sided metastatic colorectal cancer have been extensively characterized and are widely recognized as a critical prognostic factor guiding therapeutic decision-making (45, 46). In contrast, accumulating evidence indicates that adjuvant chemotherapy does not confer a consistent survival benefit in locally advanced rectal cancer, unlike its established efficacy in left-sided colon cancer (5–11). This discrepancy suggests underlying biological and clinical differences between rectal and left-sided colon tumors that may persist across both locally advanced and metastatic disease settings. Accordingly, the lack of survival benefit observed in adjuvant studies of rectal cancer provided the rationale for conducting separate analyses of treatment outcomes in metastatic rectal cancer and left-sided colon cancer (excluding the rectum).

Our analysis demonstrates that metastatic rectal adenocarcinomas are associated with a significantly more favorable prognosis compared to left-sided colon cancers. Furthermore, stratified analyses suggest that the commonly reported prognostic advantage of left-sided over right-sided colon cancer may be substantially influenced by the inclusion of rectal tumors within the left-sided cohort. Exclusion of rectal cancer cases attenuates the observed differences in clinical outcomes between left- and right-sided metastatic colon cancers. In addition, our findings indicate that targeted therapies, including anti-EGFR agents and, more prominently, anti-VEGF therapies, exhibit enhanced efficacy in metastatic rectal cancer relative to left-sided colon cancer, further supporting the biological and clinical distinction of rectal cancer as a separate entity.

A comparative analysis of gene expression in the primary tumor of RC and LSCC patients using the TCGA database revealed a group of genes with a significant difference in mRNA expression. The initial analysis included patients at all stages. Four genes (EBI3, MUC17, SOCS1, and VEGF-D) demonstrated significantly higher expression in the primary tumor of RC patients, and one gene, FAT1, showed higher mRNA expression in the primary tumor of LSCC patients. Because the main candidates for chemotherapy and targeted therapy are patients with advanced colorectal cancer, additional analysis of gene expression in the primary tumor included only patients with disease stages III-IV. In addition, a comparative analysis of the identified genes expression was conducted for patients with I-II stages and III-IV stages in both groups (RC and LSCC). Thus, it was shown that increased expression of EBI3 and SOCS1 in the RC group is determined both in the general group of patients (stages I-IV) and in the group of patients with an advanced disease (stages III-IV). Moreover, the difference in the expression of both genes increases with increasing stage of the disease. As it was previously shown, EBI3 regulates the tumor growth and antitumor cytotoxic T lymphocyte response by bidirectional reciprocal-regulation of STAT3 signaling pathway (47). The prognostic value of EBI3 for the course of colorectal cancer remains unclear, although low EBI3 expression has been shown to promote the malignant grade of gastric cancer and is associated with a poor prognosis of hepatocellular carcinoma (48, 49). Suppressors of cytokine signaling (SOCS) proteins are modulators of cytokine and growth factor signaling whose aberrant regulation have been linked to a variety of diseases. Among them, SOCS1 has been extensively investigated. Initially, it was recognized as a negative feedback regulator of cytokine signaling, for example, the Janus kinases–signal transducers and activators of transcription (JAK–STAT) signaling pathway, which is important in cellular activation, proliferation, and differentiation (40). The results of a single study conducted on 63 patients with colorectal cancer showed that SOCS1 has no prognostic value in colorectal cancer (50). However, the assessment of SOCS1 expression in this work was rather crude, using only immunohistochemical staining and defining only two groups with clearly negative and clearly positive staining. In addition, the work does not contain data of the therapy performed. As has been shown in other studies, the type of anti-tumor therapy is important because SOCS1 acts as a ferroptosis driver to inhibit a tumor progression and a platinum-based chemotherapy resistance (51). Ferroptosis, identified a decade ago, is a unique type of programmed cell death primarily driven by iron-dependent lipid peroxidation (52, 53) and playing an important role in the development of resistance to many anti-cancer drugs, including Oxaliplatin, which is widely used in the treatment of colorectal cancer (46). For breast cancer traditionally treated with platinum-based therapy, higher levels of SOCS1 expression have been shown to be associated with better clinical outcome (54, 55). While increased expression of MUC17 in the RC group loses its statistical significance in patients with the advanced disease stages III–IV, for IL-17A, on the contrary, increased expression in the RC group is significant only for patients with stages III-IV. A recent pooled meta-analysis demonstrated that IL-17A mRNA is a protective factor in colorectal cancer and a promising biomarker for assessing the prognosis and immunotherapeutic response (56). It was shown that a higher IL-17A mRNA level was associated with better overall survival rate. Furthermore, IL-17A mRNA expression also correlated positively with TNFS11, CCR6, and CCL20, indicating that the anti-tumor effect of IL-17A is likely mediated by enhancing tumor antigen presentation by dendritic cells and recruiting the activated tumor-specific CD8+ cytotoxic T lymphocytes (56). Only FAT1 mRNA demonstrated significant increase in expression in the LSCC group. FAT1 is highly expressed in most tumors. However, the role of FAT1 is still debated, being reported as a tumor suppressor (57, 58) or a tumor promoter (59). To date, there are no conclusive data on the effect of FAT1 expression on the clinical outcome of colorectal cancer.

As our analysis has shown, the RC patient group is also characterized by increased expression of VEGF family members VEGF-A, VEGF-D, and their receptor KDR-VEGFR2. Moreover, the expression level of VEGF-A and KDR-VEGFR2 directly correlates with the stage of the disease. Although KDR-VEGFR2 expression does not reach a statistically significant difference between RC and LSCC groups for patients with stages III-IV, its p-value is very close to it. Moehler, et al. (2008) (60) demonstrated that while increased, VEGF-D expression may correlate with a poorer prognosis in patients with colorectal cancer, treatment with the anti-EGFR agent cetuximab stimulated a significant decrease in VEGF-D expression, which may explain the more pronounced effect of using anti-EGFR agents in I-line therapy for RC patients.

In summary, clinical analysis indicates that the RC group experiences a markedly more favorable course of the disease after I and I+II lines of chemotherapy. This advantage may stem from higher intratumoral expression of EBI3, IL-17A, and SOCS1. The same cohort also shows greater sensitivity to anti-VEGF therapy, plausibly linked to elevated levels of VEGF-A, VEGF-D, and KDR/VEGFR2, particularly in advanced disease. It should be emphasized that the purpose of this section is not to provide a definitive biological interpretation of the identified genes, but rather to outline potential directions for future research by highlighting genes that demonstrated significant differences between the studied groups and summarizing their previously reported biological functions. In this context, the analysis should be regarded as hypothesis-generating, intended to identify candidate genes that may warrant further investigation in future mechanistic and experimental studies. Accordingly, future studies should evaluate standard and targeted regimens separately and define robust prognostic factors for right-sided colon, left-sided colon, and rectal cancers.

## Conclusion

The results of our study demonstrate that when analyzing the efficacy of antitumor therapy in colorectal cancer patients, it is necessary to distinguish not only between patients with right- and left-sided colon cancer, but also to recognize a third independent group of patients with rectal cancer. Based on these findings, it is necessary to re-evaluate treatment outcomes in metastatic colorectal-cancer patients lacking BRAF or MSI mutations, analyzing rectal cancer as a distinct subgroup. The efficacy of anti-EGFR and anti-VEGF agents in neoadjuvant chemotherapy for rectal cancer should likewise be assessed independently of their use in left- and right-sided colon cancer.

## Data Availability

The raw data supporting the conclusions of this article will be made available by the authors, without undue reservation.
