# Supplementary material for: Metastatic adenocarcinomas of left colon and rectum: differences in clinical outcome and gene expression
Source: Front Oncol. 2026 Apr 15;16:1797579. doi: 10.3389/fonc.2026.1797579 (PMC13124476; doi:10.3389/fonc.2026.1797579)
Supplement: Supplementary file 1 [file Table1.docx]

**Table S1. Patients’ parameters.**

| **Parameters** | **LSCC**  **(n=155)** | **RC**  **(n=96)** | **Р-value** |
| --- | --- | --- | --- |
| Males | 103 (66.5%) | 59 (61.5%) | 0.5 |
| Age (mean, range), years | 65.6 (34.4-86.3) | 63.3 (36.0-89.7) | 0.1 |
| ECOG  0  1  2 | 18 (11.6%)  113 (72.9%)  20 (12.9%) | 14 (14.6%)  68 (70.8%)  7 (7.3%) | 0.56  0.77  0.21 |
| Initial obstruction  None  Stoma  Stent | 102 (65.8%)  39 (25.2%)  14 (9%) | 67 (69.8%)  26 (27.1%)  3 (3.1%) | 0.79  0.77  0.077 |
| Sites of metastasis  Liver  Lungs  Lungs only  Peritoneum  Peritoneum only  Other (lymph nodes, brain, bones, ovary) | 124 (80%)  45 (29%)  12 (7.7%)  42 (27.1%)  10 (6.5%)  35 (22.6%) | 78 (81.3%)  45 (46.9%)  9 (9.4%)  13 (13,5%)  3 (3.2%)  19 (19.8%) | 0.87  **0.004**  0.81  **0.01**  0,38  0.63 |
| No. of metastatic sites  1  2  3 and more | 90 (58.1%)  48 (31%)  17 (11%) | 50 (52.1%)  36 (37.5%)  10 (10.4%) | 0.36  0.34  1 |
| No. lines of treatment  1  2  3 and more | 69 (44.5%)  55 (35.5%)  19 (12.3%) | 37 (38.5%)  35 (36.5%)  14 (14.6%) | 0.36  0.89  0.7 |
| Targeted therapy in I line  Anti-VEGF  Anti-EGFR | 63 (40.6%)  51 (32.9%) | 43 (44.8%)  34 (35.4%) | 0.6  0.78 |
| Targeted therapy in I and II lines  Anti-VEGF  Anti-EGFR  Anti-VEGF and anti-EGFR | 64 (41.3%)  32 (20.6%)  33 (21.3%) | 42 (43.8%)  22 (22.9%)  21 (21.9%) | 0.79  0.75  1 |
| Palliative radiation therapy | 12 (12.5%) | 1 (0.6%) | **<0.0001** |
| CEA> 5,0 ng/ml  Mean when > 5,0 ng/ml | 65/81 (80.2%)  885 | 28/36 (77.8%)  292 | 0.81  0.068 |
| СА 19-9> 30 IU/ml  Mean when > 30 IU/ml | 39/77 (50.6%)  1928 | 21/35 (60%)  317 | 0.42  **0.007** |
| RASwt  Unknown | 81 (61.4%)  23 (14.8%) | 50 (55.6%)  6 (6.3%) | 0.41 |
| BRAF mut  Unknown | 6 (4.7%)  27 (17.4%) | 2 (2.3%)  8 (8.3%) | 0.48 |
| MSI  Unknown | 1 (0.76%)  26 (16.8%) | 3 (3.4%)  9 (9.4%) | 0.31 |

**Table S2. Characteristics of the TCGA database patients used for the gene expression analysis.**

| **Parameters** | **LSCC (n=153)** | **RC (n=41)** | **P-value** |
| --- | --- | --- | --- |
| **Sex:** |  |  |  |
| Males | 77 (50.3%) | 24 (58.5%) | 0.35 |
| Females | 76 (49.7%) | 17 (41.5%) | 0.35 |
| **Race:** |  |  |  |
| White | 119 (83.8%) | 29 (90.6%) | 0.26 |
| Black | 19 (13.4%) | 2 (6.3%) | 0.33 |
| Asian | 3 (2.1%) | 1 (3.1%) | 0.73 |
| Amer. Indian or Alaska Native | 1 (0.7%) | 0 (0%) | 0.63 |
| **Age** (mean, range), years | 62.4 (36-90) | 63.7 (33-90) | 0.55 |
| **AJCC Pathologic Tumor Stage:** |  |  |  |
| Stage 1 | 21 (14.6%) | 4 (10%) | 0.52 |
| Stage II | 53 (36.8%) | 15 (37.5%) | 0.94 |
| Stage III | 46 (31.9%) | 15 (37.5%) | 0.51 |
| Stage IV | 24 (16.7%) | 6 (15%) | 0.8 |

**Table S3. Univariate logistic regression analysis.**

| **Factors** | **n** | **OS (months)** | **Р-value (RR; 95% CI)** |
| --- | --- | --- | --- |
| Sex:  Male  Female | 162  89 | 20,8  19,6 | 0,92  (1,02; 0,74-1,4) |
| Age:  <65 years  >65 years | 121  130 | 24,3  17,6 | **0,005**  **(1,55; 1,14-2,11)** |
| ECOG  0-1  2 | 213  27 | 21,1  6,6 | **0,019**  **(1,75; 1,1-2,78)** |
| Localization:  LSCC  RC | 155  96 | 16,2  25,8 | **<0,0001**  **(0,52; 0,38-0,73)** |
| Localization of metastases*  Lungs  Peritoneum | 81  45 | 22,1  14,1 | 0,15  (1,39; 0,89-2,18) |
| Number of affected organs  1-2  >3 | 224  27 | 21,9  14,7 | 0,15  (1,41; 0,88-2,25) |
| CEA  <5 ng/ml  >5 ng/ml | 24  93 | 17,4  19,5 | 0,79  (0,93; 0,54-1,6) |
| CA 19-9  <30 units/L  >30 units/L | 52  60 | 24,4  17,0 | 0,2  (1,37; 0,85-2,22) |

* Patients with metastases in both the peritoneum and the lungs are not included

# **Table S4. Multivariate logistic regression analysis.**

|  | **B** | **Standard Error** | **Wald** | ***df*** | **Sig.** | **Exp(B)** | **95% Confidence Interval for Exp(B)** | |
| --- | --- | --- | --- | --- | --- | --- | --- | --- |
|  |  |  |  |  |  |  | **Lower Bound** | **Upper Bound** |
| Age | 0,431 | 0,161 | 7,153 | 1 | 0,007 | 1,538 | 1,122 | 2,109 |
| ECOG | 0,576 | 0,237 | 5,929 | 1 | 0,015 | 1,780 | 1,119 | 2,830 |
| Localization | -0,587 | 0,174 | 11,406 | 1 | 0,001 | 0,556 | 0,396 | 0,782 |

**Table S5. Progression-free survival of I and I+II lines of therapy and overall survival**

| **Groups** | **n** | **PFS**  **I line** | **Р-value**  **(HR, 95%CI)** | **PFS**  **I+II line** | **Р-value**  **(HR, 95%CI)** | **OS** | **Р-value**  **(HR, 95%CI)** |
| --- | --- | --- | --- | --- | --- | --- | --- |
| **LSCC** | 155 | 7.4 m. | 0,002  HR 1,53  1.17-2.0 | 11.5 m. | <0,0001  HR 1,76  1,3-2.4 | 16.2 m. | <0,0001  HR 1.91  1.37-2.66 |
| **RC** | 96 | 9.9 m. |  | 17.2 m. |  | 25.8 m |  |

**Table S6. I line PFS and OS analysis for RSCC, LSCC, RC, and LSCC+RC groups of patients.**

| **Type** | **n** | **PFS** | **Р-value** | **OS** | **Р-value**  **(HR, 95%CI)** |
| --- | --- | --- | --- | --- | --- |
| **RSCC** | 56 | 9.3 | 0.34 | 17.8 | 0.343 |
| **LSCC** | 63 | 7.8 | Ref | 17.9 | Ref |
| **RC** | 43 | 10.5 | 0.02 | 25.8 | 0.016 |
| **LSCC+RC** | 106 | 8.9 | 0.77 | 21.9 | 0.027 |
